# N-Acetylcysteine or Sodium Selenite Prevent the p38-Mediated Production of Proinflammatory Cytokines by Microglia during Exposure to Mercury (II)

**DOI:** 10.3390/toxics10080433

**Published:** 2022-07-29

**Authors:** Vasco Branco, Lucia Coppo, Michael Aschner, Cristina Carvalho

**Affiliations:** 1 Research Institute for Medicines (iMed.ULisboa), Faculty of Pharmacy, Universidade de Lisboa, 1649-003 Lisbon, Portugal; 2 Centro de Investigação Interdisciplinar Egas Moniz (CiiEM), Instituto Universitário Egas Moniz (IUEM), 2829-511 Caparica, Portugal; 3 Division of Biochemistry, Department of Medical Biochemistry and Biophysics, Karolinska Institute, SE-171 77 Stockholm, Sweden; lucia.coppo@ki.se; 4 Department of Molecular Pharmacology, Albert Einstein College of Medicine, Bronx, NY 10461, USA; michael.aschner@einsteinmed.edu

**Keywords:** mercury, p38, microglia, inflammation, thioredoxin reductase, glutathione, selenium, N-acetylcysteine

## Abstract

Mercury (Hg) is known for its neurotoxicity and is reported to activate microglia cells at low exposure levels. Since mercury decreases the activity of the glutathione and thioredoxin systems, we hypothesize that Hg would, in turn, disrupt microglia homeostasis by interfering with redox regulation of signaling pathways. Thus, in this work, we analyzed the effect of exposure to Hg^2+^ on nuclear translocation and activation of NF-kB (p50) and p38 and pro-inflammatory gene transcription (IL-1ß; iNOS, TNF-alpha) considering the interaction of Hg with the glutathione system and thioredoxin systems in microglial cells. N9 (mouse) microglia cells were exposed to different concentrations of Hg^2+^ and the 24 h EC_50_ for a reduction in viability was 42.1 ± 3.7 μM. Subsequent experiments showed that at sub-cytotoxic levels of Hg^2+^, there was a general increase in ROS (≈40%) accompanied by a significant depletion (60–90%) of glutathione (GSH) and thioredoxin reductase (TrxR) activity. Upon 6 h of exposure to Hg^2+^, p38 (but not p50) accumulated in the nucleus (50% higher than in control), which was accompanied by an increase in its phosphorylation. Transcript levels of both IL1-ß and iNOS were increased over two-fold relative to the control. Furthermore, pre-exposure of cells to the p38 inhibitor SB 239063 hindered the activation of cytokine transcription by Hg^2+^. These results show that disruption of redox systems by Hg^2+^ prompts the activation of p38 leading to transcription of pro-inflammatory genes in microglia cells. Treatment of N9 cells with NAC or sodium selenite—which caused an increase in basal GSH and TrxR levels, respectively, prevented the activation of p38 and the transcription of pro-inflammatory cytokines. This result demonstrates the importance of an adequate nutritional status to minimize the toxicity resulting from Hg exposure in human populations at risk.

## 1. Introduction

Mercury (Hg) compounds are well-established neurotoxicants [1]. The ability to cross the blood–brain barrier is a key feature explaining the tropism of these compounds to the central nervous system (CNS) [2]. Regardless of the exposure source (fish, thimerosal-containing vaccines or mercury vapor) and the compound in question (methylmercury, ethylmercury, elemental mercury vapor), Hg will accumulate in the CNS, and once a threshold is reached, toxicity will ensue, characterized by motor and cognitive function deficits which are especially pernicious during in utero development [3].

Immunotoxic effects of Hg are reported to be triggered at an exposure level below the neurotoxicity threshold. In this sense, microglial cells—the major representatives of innate immunity in the CNS—are potential targets of early toxicity by mercury compounds. Indeed, microglia activation by mercury has been shown to occur at very low (<1 μM) levels of exposure [4]. Recently, Tan and co-workers [5] reported that Hg^2+^ enhanced microglial cytokine production via ERK phosphorylation and an earlier study with murine macrophages showed that Hg^2+^ increases p38 activation at sub-cytotoxic levels [6].

Mercury compounds target nucleophilic sites of peptides and proteins, namely thiol (SH-) and selenol (SeH-) groups in cysteine (Cys) and selenocysteine (Sec) residues, respectively [7]. These reactive residues are key for the activity of several enzymes, such as thioredoxin (Trx), thioredoxin reductase (TrxR) and glutaredoxin, which regulate redox signal transduction [8]. Since regulation of the pathways involved in cytokine production requires redox signaling through Cys switches [9], we hypothesized that Hg would disrupt microglial homeostasis by interfering with the activity of redox systems.

Both the thioredoxin and glutathione/glutaredoxin systems are established targets for all Hg compounds, with Hg^2+^, being the most potent inhibitor [10]. In particular, inhibition of the selenoenzyme TrxR is known to occur at levels below the cytotoxicity threshold due to the reactivity of Sec residue in its active site [10,11]. Attempts to counteract this inhibition using selenium supplementation have provided mixed results since the Se levels used were over the physiological essentiality threshold which in the long term could generate prooxidant effects [12,13]. A reasonable alternative would be to optimize the cellular selenium status by supplementing cellular media with a sufficient amount of sodium selenite to optimize selenoprotein levels [14,15]. In the case of human populations, this would entail assuring a proper nutritional intake of Se-rich foods [16].

In this work we aimed to disclose the effect of the interaction between Hg^2+^ and redox systems in microglia cells, examining TrxR activity and GSH concentration, p38 and NF-kB activation and cytokine production. Moreover, we addressed the protective effect of N-acetylcysteine (NAC) and sodium selenite supplementation on Hg^2+^ toxicity in microglia.

## 2. Materials and Methods

### 2.1. Cell Culture

N9 mouse microglia were a kind gift from Professor Dora Brites (iMed.ULisboa). Cells were cultured in RPMI 1640 medium (Gibco, Waltham, USA) supplemented with 10% FBS (Biochrom AG, Berlin, Germany) and 1% Pen-Strep Mix (Gibco). Cells were kept at 37 °C in a humidified incubator under a 5% CO_2_ atmosphere. For passing, plating or collection of cells from plates, the medium was removed, and the cells were rinsed with Versene (Gibco) and incubated at 37 °C with 0.05% Trypsin-EDTA (Gibco) for 10 min, after which culture medium was added to inhibit trypsin.

### 2.2. Cell Viability

Cell viability was determined by the MTT assay as initially described by [17] (and subsequently modified [13]). Cells (5 × 10^3^ cells/well) were seeded in 96-well plates and after 24 h various concentrations (range: 0–50 μM) of HgCl_2_ (from Sigma-Aldrich, St Louis, USA; hereafter referred as Hg^2+^) were added to plates and the exposure lasted 24 h, 48 h and 72 h. Subsequently, MTT was added to each well at a final concentration of 400 μg/mL per well, followed by incubation at 37 °C for 2 h. Following the incubation period, the MTT solution was removed, formazan crystals were dissolved with 4:1 dimethyl sulfoxide/glycine solution (pH 10.5) and the absorbance was recorded at 550 nm. The EC_50_ was calculated as the compound concentration causing a 50% decrease in MTT reduction relative to the control group. In subsequent experiments, 10 μM was the maximum Hg^2+^ concentration used, as up to this level no significant change in cellular viability was observed after 24 h. This concentration was also consistent with literature reports on post-mortem levels of Hg^2+^ in humans [18,19,20,21].

### 2.3. Reactive Oxygen Species (ROS)

Intracellular ROS levels were determined as previously described [22], using 2′-7′-dichlorodihydrofluorescein diacetate (DCFH-DA;Sigma-Aldrich, St Louis, USA). Briefly, cells were seeded (8 × 10^4^ cells/well) for 24 h in black 96-well plates and incubated with a medium containing 25 μM DCFH-DA for 45 min, at 37 °C. After this period, DHCF was removed, and the cells were rinsed twice with PBS prior to the addition of a fresh culture medium containing different concentrations of Hg^2+^ (0–10 μM;). Wells without cells were used to check background fluorescence and cells exposed to 200 μM H_2_O_2_ (1 h) were used as a positive control. After 24 h of exposure, fluorescence was detected in a multi-well plate reader, using 485 nm and 530 nm excitation and emission wavelengths, respectively. Procedures were performed under low light conditions to avoid DHCF degradation.

### 2.4. Preparation of Cell Lysates

Cells were seeded in 10 mm^2^ culture dishes (1 × 10^6^ cell/plate) and allowed to reach 70–80% confluency. Then, the culture medium was renewed and cells were exposed to Hg^2+^ (0, 1, 5 and 10 μM). Exposure time and cell lysis were carried out according to the specificities of each analysis (see subsequent sections).

### 2.5. Total Protein Determination

Total protein was quantified in lysates by mixing each sample with diluted (5×) Coomassie dye (Bio-Rad, Hercules, CA, USA) in 96-well plates as described by Bradford [23]. Absorbance was measured at 595 nm in a microplate reader (Zenyth 3100, Anthos Labtec Instruments, Salzburg, Austria) and the protein concentration was calculated from a calibration curve using bovine serum albumin as the standard.

### 2.6. Total Glutathione Quantification

Total GSH was determined using the DTNB reduction method described by [24] as subsequently detailed. Following exposure (24 h), cells were resuspended in cold-ice extraction buffer (0.1% Triton X-100 and 0.6% sulfosalicylic acid in 0.1 M potassium phosphate buffer with 5 mM EDTA disodium salt, pH 7.5, all from Sigma-Aldrich, St. Louis, USA) and sonicated for 3 min. Following centrifugation at 3000× *g* for 5 min at 4 °C, the supernatant was used for GSH measurements. Briefly, 5 μL of each sample were incubated with 1 mM DTNB, 200 μM NADPH, and 50 nM glutathione reductase (100 U/mL GR) in 0.1 M potassium phosphate buffer with 5 mM EDTA (pH 7.5) with 5′-thio-2-nitrobenzoic acid (TNB) formation being followed at 412 nm for 2 min. Calibration curves (range: 0.5–50 μM) were used for quantification and GSH levels were normalized for protein level and expressed relative to control. Quantification of cells exposed for 24 h to BSO (100 μM) was used as a positive control for glutathione depletion.

### 2.7. Thioredoxin Reductase Activity

Thioredoxin reductase activity was measured in total cell lysates (24 h exposure) by the insulin endpoint assay for complex biological samples as described by Arnér and Holmgren [25]. Briefly, samples (30 μg of protein) were incubated in 96-well plates with 0.3 mM insulin, 660 μM NADPH, 3 mM EDTA, and 1 μM of reduced human Trx1 (IMCO Corp., Stockholm, Sweden), in 85 mM Hepes buffer (pH 7.6) at 37 °C for 20 min. In parallel, wells containing the same reagents but excluding Trx addition were prepared. After the incubation period, the reaction was stopped with a DTNB/guanidine-HCl solution (pH 8.0). Absorbance was measured in a microplate reader (Zenyth3100, Anthos Labtec Instruments, Salzburg, Austria) at 412 nm, and TrxR activity was quantified as the difference in absorbance between the Trx containing well and the corresponding well without Trx.

### 2.8. Nuclear Expression of p38 and p50

Microglia cells were exposed for 6 h to Hg^2+^ (5 μM) as described above with or without pre-exposure to NAC (2.5 mM; 24 h) or sodium selenite (0.1 μM, 24 h). Afterward, for the collection of nuclear extracts, cells were trypsinized (0.05% trypsin-EDTA), washed in PBS, centrifuged and the cellular pellet resuspended in hypotonic buffer (10 mM HEPES pH 7.9, 1.5 mM MgCl_2_, 10 mM KCl, 0.5 mM DTT and protease inhibitor cocktail) followed by incubation on ice for 15 min. NP-40 (0.5%) was added and cells were vortex-mixed for 10 s prior to the recovery of nuclear pellets by centrifugation (6500× *g*, 20 s). After further washing with hypotonic buffer and centrifugation (6500× *g* for 20 s), the nuclear pellet was resuspended in a high salt lysis buffer (20 mM HEPES pH 7.9, 1.5 mM MgCl_2_, 420 mM NaCl, 0.2 mM EDTA, 25% glycerol and protease inhibitor cocktail), stirred for 30 min at 4 °C and centrifuged for 10 min at 13,000× *g* and 4 °C and the supernatants (i.e., the nuclear extracts) collected [13]. After total protein determination in nuclear extracts, expression of p50, p38 and phosphorylated p38 was evaluated by western blot with the following antibodies anti- p50 mouse monoclonal IgG (sc-13032, H300); anti-p38α mouse monoclonal IgG (sc-136210); anti-phospho-p38 (sc166182; E1) all from Santa Cruz Biotechnology Inc, USA. Following washing and incubation with appropriate secondary HRP-conjugated antibodies, expression was detected by chemiluminescence in a Chemidoc station, band intensity quantified using the Chemidoc Software (Bio-Rad) and levels normalized for the total loading using Ponceau S staining (Sigma-Aldrich, St. Louis, USA).

### 2.9. Expression of Pro-Inflammatory Cytokines

To evaluate the effects over pro-inflammatory gene transcription, N9 cells were exposed to different treatments: (i) control (Ct); (ii) positive control (LPS; 300 ng/mL, for 24 h); (iii) Hg^2+^ (5 μM; 24 h); (iv) pre-exposure (24 h) to NAC (2.5 mM), followed by media change and exposure to Hg^2+^ (5 μM; 24 h); (v) pre-exposure (24 h) to sodium selenite (0.1 μM) followed by media change and exposure to Hg^2+^ (5 μM; 24 h); (vi) pre-exposure (4 h) to the p38 inhibitor SB 239063 (100 μM) followed by media change and exposure to Hg^2+^ (5 μM; 24 h).The SB 239063 has high specificity for the p38α isoform, with a significantly lower IC_50_ relative to other protein kinases [26]. The concentration used here is in line with previous reports in microglial cells [27]. Groups with pre-exposure to NAC, Se and SB 239063 but without subsequent exposure to Hg^2+^, were also performed as controls (data not shown). NAC and sodium selenite concentrations were selected based on previous reports in the literature [28,29].

After exposure, RNA extraction was performed with NZY Total RNA Isolation kit (NZYTech, Lisbon, Portugal) and cDNA synthesized with NZY First-Strand cDNA Synthesis Kit (NZYTech, Portugal) according to the manufacturer’s protocols. Quantification of interleukin-1ß (IL1-ß), Nitric oxide synthetase 2 (iNOS) and Tumor Necrosis Factor alpha (TNF-α) transcript expression was conducted by qPCR using GAPDH as the housekeeping gene and the 2^−∆∆CT^ method for relative quantification [30]. The primers used are described in Table 1. Primer efficiency was assessed by testing serial dilutions (1:10 to 1:1,000,000) of the cDNA template and varied between 95% and 108%.

PCR reactions contained NZY qPCR Green Master Mix with ROX as the passive dye (NZYTech), 400 nM forward primer, 400 nM reverse primer, and 100 ng of template cDNA in a final 20 μL volume. The initial incubation was set at 50 °C for 2 min and 95 °C for 10 min and was followed by 40 cycles of amplification (15 sec at 95 °C plus 1 min at 60 °C). Reactions were performed in an Applied Biosystems QuantStudio^TM^ 7 Flex Real-Time PCR System (Applied Biosystems, Foster City, CA, USA).

### 2.10. Statistical Analysis

Results are presented as mean ± standard error (S.E.) of three to five independent experiments. Differences between groups were evaluated using either a One-Way ANOVA followed by Dunnet’s multiple comparison test or with Student’s t-test for independent samples. Differences were considered significant at *p* < 0.05 and very significant at *p* < 0.01.

## 3. Results

### 3.1. Cell Viability

The effect of Hg^2+^ exposure on cellular viability showed a time- and concentration-dependent profile (data not shown), with EC_80_ and EC_50_ for a decrease in MTT reduction after 24 h exposure of 21.8 μM and 45.0 μM, respectively. Based on these results, and since we aimed to carry out the studies in the sub-cytotoxic range, 10 μM was the maximal concentration used in subsequent experiments since up to this value no significant change in cellular viability was noted after 24 h.

### 3.2. Thioredoxin Reductase Activity, Total Glutathione Levels and ROS Production

**Figure 1:**
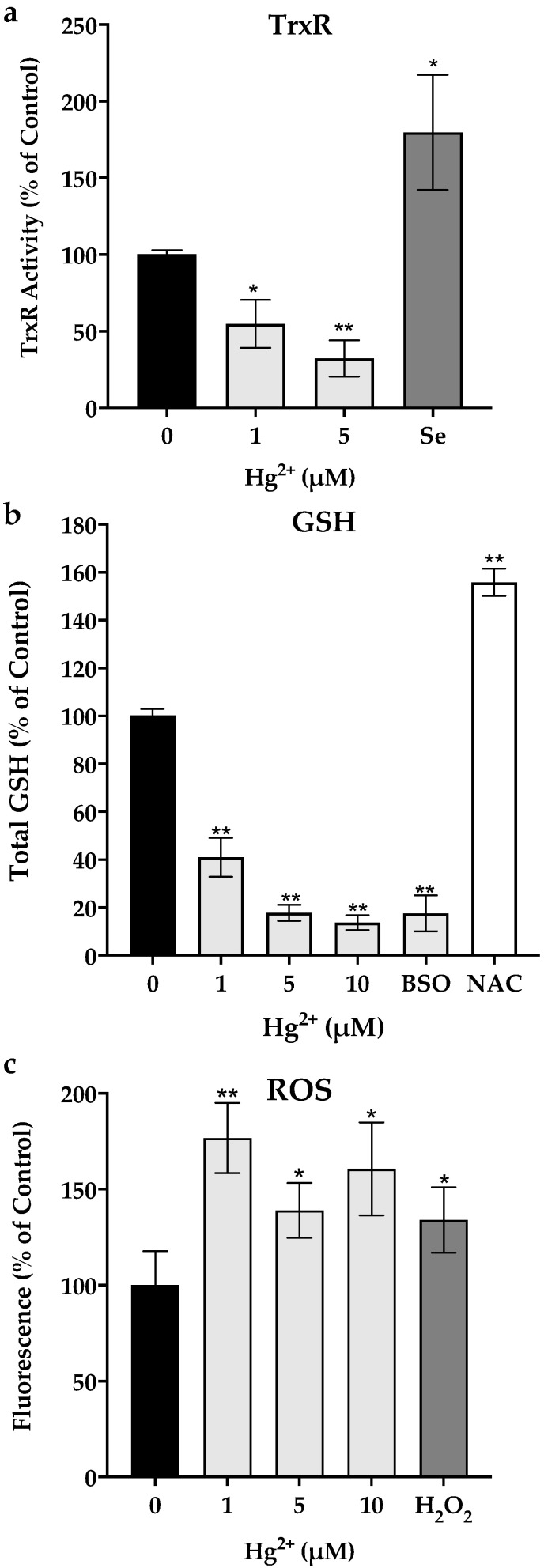
Activity of thioredoxin reductase (TrxR, **panel a**), total glutathione (GSH, **panel b**) and reactive oxygen species levels (ROS; **panel c**) in N9 cells exposed for 24 h to different concentrations of Hg^2+^. Exposure to sodium selenite (0.1 μM) for 24 h was used to evaluate effects over TrxR activity. Exposure to NAC and BSO was used to evaluate the effect of upregulation and depletion on GSH levels. Data represent the mean ± S.E. of 3–5 independent experiments. * significantly different from control (*p* < 0.05); ** very significantly different from control (*p* < 0.01).

Following 24 h of exposure to Hg^2+^, the TrxR activity of mouse microglial cells was significantly decreased to ~30% of control activity at an exposure concentration of 5 μM (Figure 1a). Likewise, total GSH levels were significantly reduced in a concentration-dependent manner (Figure 1b). The decrease in TrxR and GSH was accompanied by an increase in ROS production as seen in the DHCF assay (Figure 1c).

On the other hand, exposure of N9 microglia cells to 0.1 μM of sodium selenite up-regulated TrxR activity up to two-fold relatively to the control. Similarly, pre-exposure of cells to NAC (2.5 mM) increased GSH levels by 50% relative to the control group.

### 3.3. Effect of Hg^2+^ on p50 and p38 Nuclear Levels

**Figure 2:**
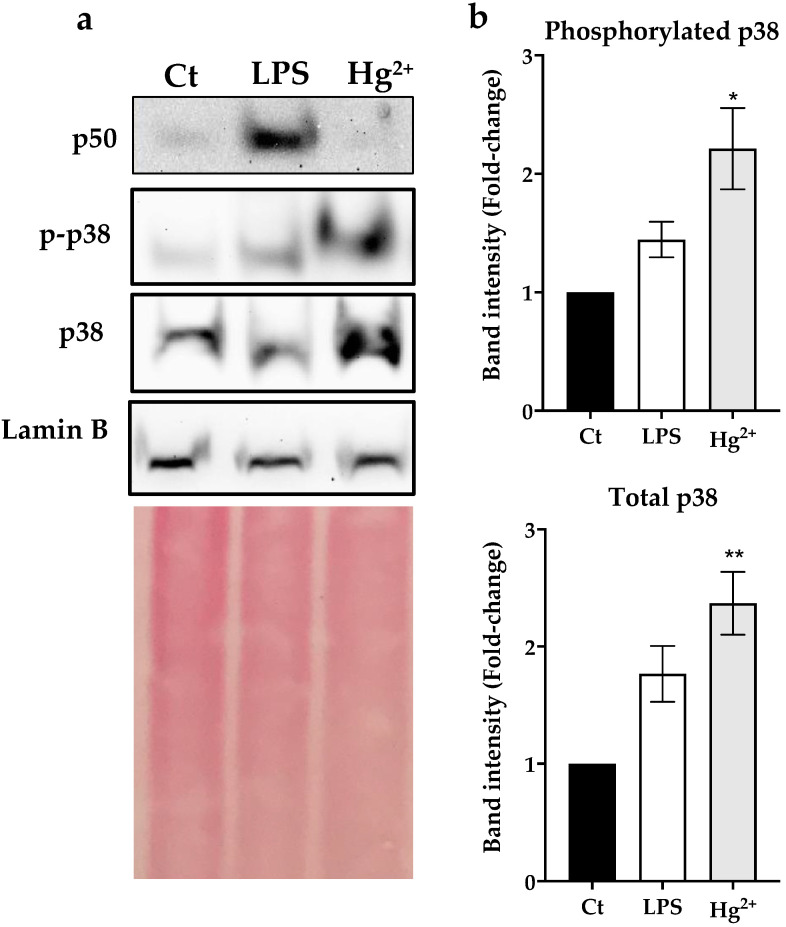
Nuclear levels of p50, p38 and phosphorylated p38 in the nuclear fraction of N9 cells exposed for 6 h to Hg^2+^ (5 μM) or Lipopolysaccharide (LPS; 300 ng/mL). (**a**) Western blot results for p50 (50 kDa), p38 (38 kDa) and phosphorylated p38 (p-p38; 38 kDa) using Lamin B (67 kDa) as a nuclear marker. (**b**) Band intensity analysis for p38 and p-p38. Data represent the mean ± S.E. of 3 independent experiments. * significantly different (*p* < 0.05) from non-treated control (Ct); ** very significantly different (*p* < 0.01) from non-treated control (Ct).

Exposure of N9 cells for 6 h to 5 μM Hg^2+^ significantly increased p38 translocation to the nucleus (Figure 2). The phospho-p38/p38 ratio was slightly higher in the Hg^2+^ (0.92) relative to the control group (0.85), but there was a clear three-fold increase in the amount of phosphorylated p38, pointing to increased activation of this pathway. On the other hand, no nuclear translocation of p50 was observed.

### 3.4. mRNA Levels of IL1-ß, TNF-α and iNOS

**Figure 3:**
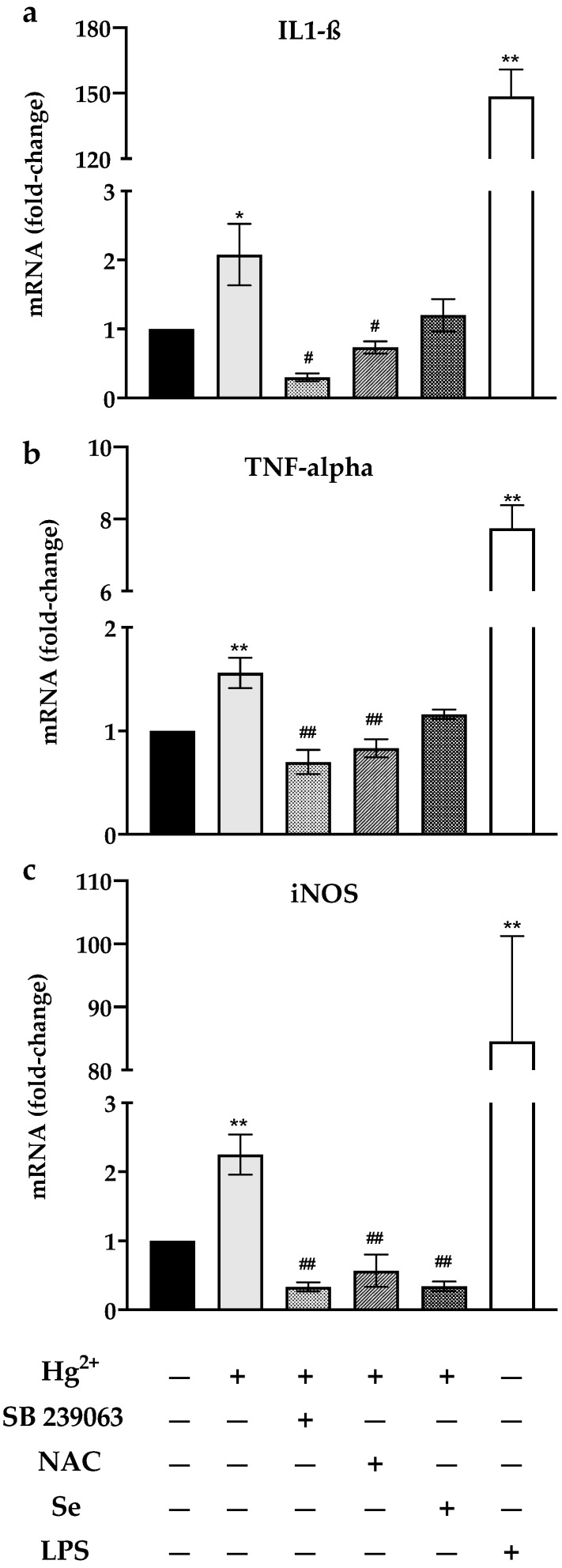
Transcript (mRNA) levels for IL1-ß (**a**), TNF-alpha (**b**) and iNOS (**c**) following exposure of N9 cells for 24 h to Hg^2+^ (5 μM) alone or after pre-exposure to either the p38 inhibitor SB 239063 (4 h, 100 μM), N-acetylcysteine (NAC; 2.5 mM, 24 h) or sodium selenite (Se; 0.1 μM, 24 h). Cells exposed to Lipopolysaccharide (LPS) were used as a positive control. Data represent the mean ± S.E. of 3–5 independent experiments. * significantly different (*p* < 0.05) from non-treated control (Ct); ** very significantly different (*p* < 0.01) from Ct; ^#^ significantly different (*p* < 0.05) from cells treated with Hg^2+^; ^##^ very significantly different (*p* < 0.01) from cells treated with Hg^2+^.

When N9 cells were exposed to Hg^2+^ (5 μM; 24 h) there was an approximately two-fold increase in the transcript levels of IL1-ß, TNF-α and iNOS (Figure 3a–c, respectively). Interestingly, pre-exposure of cells to sodium selenite (0.1 μM, 24 h) or NAC (2.5 mM; 24 h) had the same effect as the p-38 inhibitor (SB 239063; 4 h), fully preventing the up-regulation of mRNA for these three genes upon exposure to Hg^2+^ (Figure 3).

### 3.5. Effect of Pre-Exposure to NAC and Selenite over p38 Activation by Hg^2+^

**Figure 4:**
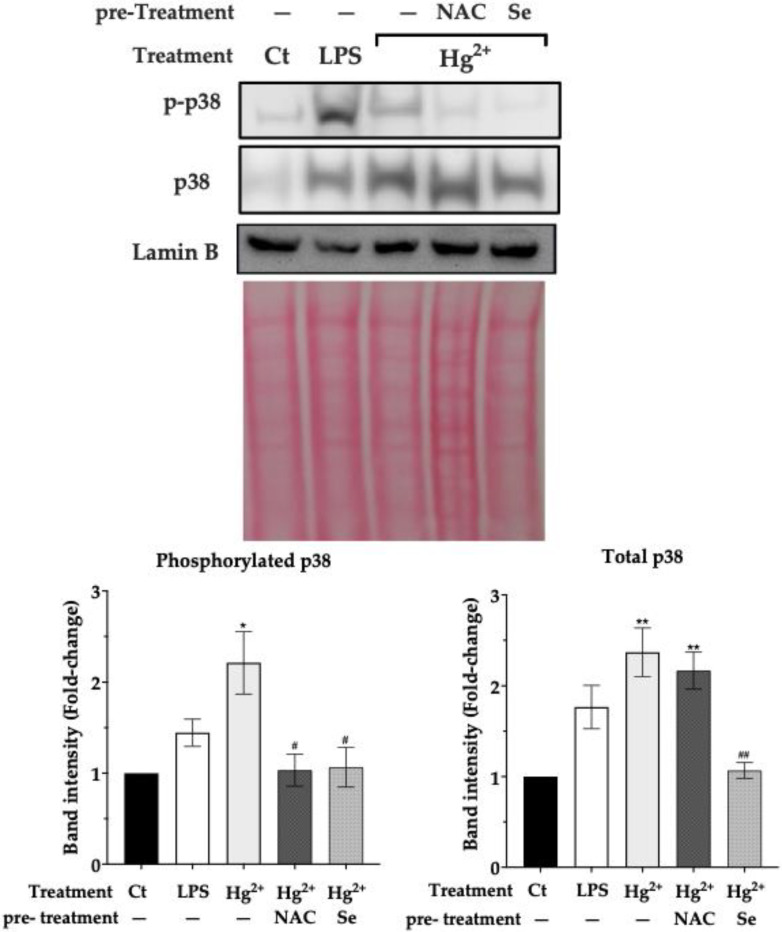
Nuclear levels of p38 and phosphorylated p38 in the nuclear fraction of N9 cells after 6 h of exposure to Hg^2+^ (5 μM) with and without pre-exposure (24 h) to NAC (2.5 mM) or sodium selenite (0.1 μM). LPS—Lipopolysaccharide (300 ng/mL). Data represent the mean ± S.E. of 3 independent experiments. * significantly different (*p* < 0.05) from non-treated control (Ct); ** very significantly different (*p* < 0.01) from Ct; ^#^ significantly different (*p* < 0.05) from cells treated with Hg^2+^; ^##^ very significantly different (*p* < 0.01) from cells treated with Hg^2+^.

Figure 4 shows the effect of pre-exposure (24 h) of N9 cells to NAC (2.5 mM) or sodium selenite (0.1 μM) on the nuclear translocation and phosphorylation of p38 by Hg^2+^. Interestingly, NAC did not reduce significantly p38 translocation but did prevent its phosphorylation, i.e., activation. Sodium selenite pre-exposure reduced both nuclear accumulation of p38 and activation after 6 h of exposure to Hg^2+^.

## 4. Discussion

Mercury (Hg) compounds are soft electrophiles that target thiols and selenols in Cys and Sec residues, respectively [3]. These amino acid residues are highly reactive, and therefore, have a prominent role in regulating redox-dependent signaling circuits, such as MAPKs or transcription factor activation and DNA binding [7].

Here, we show that Hg^2+^ at sub-cytotoxic levels promptly decreases the activity of TrxR, a selenoprotein, and reduces the levels of the most abundant antioxidant cellular thiol, GSH. The TrxR inhibition observed herein in N9 microglial cells (Figure 1a) was comparable to our previous reports [10,13] in other cell lines, corroborating the sensitivity of this enzyme to Hg^2+^. Likewise, GSH levels (Figure 1b) were significantly diminished by the exposure to Hg^2+^, corroborating previous studies [31,32]. Because of the significant reduction in the activity of the two major redox systems, it is, therefore, not surprising that ROS levels increased accordingly (Figure 1c).

Concomitantly to the downregulation of TrxR activity and GSH, we observed an augmented translocation of p38 to the nucleus alongside an increase in its phosphorylation (Figure 2). Phosphorylation of p38 at conserved tyrosine and threonine residues activates its kinase function [33]. The p38 MAPK plays an important role in several signaling cascades, such as inflammation, regulating the transcription of several important genes [34]. This MAPK is known to be regulated by redox mechanisms and GSH depletion has been shown to cause its activation [35], leading to enhanced transcription or pro-inflammatory genes.

Interestingly, we failed to observe translocation of NF-kB p50 subunit to the nucleus in N9 cells exposed to Hg^2+^ (Figure 2). NF-kB is the transcription factor responsible for a canonical response to TLR4-activating endotoxins, such as LPS [36]. Indeed, when we exposed cells to LPS as a positive control, we could observe an augmented translocation of p50 to the nucleus. Curiously, previous studies showed that Hg^2+^ can interfere with the responsiveness of cells to LPS, leading to diminished activation of NF-kB, but not activating the pathway itself [6]. Indeed, Hg^2+^ unlike other metals, such as nickel or gold, does not activate TLR3/4 receptors [37], and therefore, p38 appears to be a key regulator of Hg-induced inflammatory responses [6].

Our experiments showed that Hg^2+^ leads to an increase in the mRNA levels of pro-inflammatory cytokines (IL1-ß; TNF-α) and iNOS (Figure 3). When cells were pre-exposed to the p38 inhibitor, SB239063, followed by exposure to Hg^2+^, mRNA levels were substantially decreased relatively to control, corroborating the role of p38 in mercury-induced transcription of IL1-ß, TNF-α and iNOS. Interestingly, a previous study reported a predominance of ERK and STAT3 signaling versus p38 signaling in N9 cells exposed to Hg^2+^ [5]. However, the authors used either a very low concentration of Hg^2+^ (15 ng/mL; approx. 75 nM) that promoted cell survival and that is unlikely to negatively affect the redox status of cells, or a very high concentration (15 μg/mL, approx. 75 μM) that induces cytotoxicity. Our experiments used an intermediate exposure level that seems to affect both redox systems, and therefore, upregulates p38 signaling. Hence, depending on the exposure dose, different kinase pathways could be activated or inhibited by Hg^2+^.

Additionally, our experiments showed that N9 cells were protected from inflammatory signaling when exposed to either NAC or sodium selenite prior to being exposed to Hg^2+^. Pre-exposure to NAC increased cellular GSH levels by 50% relative to the control (Figure 1b), since NAC is a precursor to Cys, which is the limiting substrate for GSH synthesis [28]. This pre-exposure completely prevented the transcription of IL1-ß, TNF-α and iNOS genes by Hg^2+^ (Figure 3). Moreover, pre-exposure to NAC reduced p38 phosphorylation caused by Hg^2+^ (Figure 4). This is in agreement with results showing that GSH down-regulates TNF-α production via p38 signaling in bronchial epithelial cells [35] and with NAC’s anti-inflammatory action [38], which encompasses an inhibitory effect over p38 [39].

Likewise, pre-exposure of cells to sodium selenite (0.1 μM) increased TrxR activity (Figure 1a) and prevented the transcription of pro-inflammatory genes as well (Figure 3). TrxR, being the major regulator of thioredoxin activity, is involved in the suppression of the MAP3K Apoptosis Signaling Kinase 1 (ASK-1) by thioredoxin. ASK-1 is upstream of an oxidative stress sensitive cascade regulated by thioredoxin’s redox state and culminating in p38 activation [40]. As we have shown previously [41], by inhibiting the thioredoxin system, mercury compounds cause the activation of ASK-1. Herein, sodium selenite supplementation causing TrxR up-regulation prevented p38 activation (Figure 4) by presumably acting upstream on ASK-1 via Trx. Notably, the beneficial effects of selenite occurred at a low dose (0.1 μM), well below the doses normally reported in experiments addressing the effect of Se on Hg toxicity (see for example [13]). In fact, using a high dose of selenite to achieve a Se:Hg molar ratio > 1 may counterproductively lead to further toxicity due to the redox cycling of selenide (Se^2−^) with oxygen (see [7] for details). Similarly, studies using a selenite level in line with our experiments (100 nM) also show a reduction in p38 signaling [42], whereas studies using higher levels (>1 μM) show activation of this pathway [43]. Thus, it is better to use relatively low selenite supplementation which is sufficient to up-regulate the levels of selenoproteins, particularly TrxR, and mitigate Hg^2+^ pro-inflammatory effects by blocking p38 activation. In fact, selenite supplementation was shown to be more effective than NAC since it more evidently prevented p-38 activation (Figure 4) and inflammatory gene transcription (Figure 3).

It should be stressed that regardless of the selenium form present in food and the differences in bioavailability between compounds [44], once absorbed, selenium compounds are reduced to selenide (Se^2−^) and integrated into selenoprotein synthesis [7]. In the liver, part of this dietary selenium is used to synthesize selenoprotein P which is then released into the bloodstream and functions as a major selenium-delivery system to the different organs, including the brain [45].

**Figure 5:**
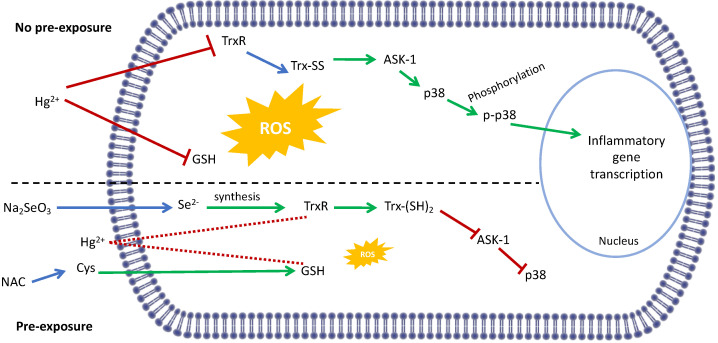
Proposed mechanism of p38 activation by Hg^2+^ and selenite and NAC protection. Exposure to Hg^2+^ reduces GSH levels and TrxR activity leading to the production of ROS and oxidation of Trx which activates the MAP3K ASK-1 and downstream p38. This is phosphorylated and translocates to the nucleus where it promotes inflammatory gene transcription. If cells are pre-exposed to NAC or sodium selenite, GSH or TrxR levels are maintained, the inhibitory effect of Hg^2+^ mitigated (dotted lines) and no p38 activation is observed. Molecular pathways and signaling cascades are simplified for clarity. Green arrows indicate activation of a pathway and red lines inhibition. Blue arrows indicate links between TrxR inhibition and Trx oxidation, selenite supplementation and Se^2−^ availability and NAC supplementation and Cys availability.

Overall, our novel results can be summarized as shown in Figure 5. Exposure to Hg^2+^ causes a decrease in TrxR activity and available GSH leading to ROS production and presumably to activation of ROS-sensitive ASK-1. Reduced Trx (Trx-(SH)_2_) is known to suppress ASK-1 and its oxidation (Trx-SS), readily releases ASK-1 and initiates a signaling cascade activating p38 which translocates to the nucleus where it promotes inflammatory gene transcription. On the other hand, if either TrxR or GSH levels are increased by selenite or NAC pre-treatment, respectively, redox homeostasis is maintained, and inflammatory gene transcription is not activated.

## 5. Conclusions

These results provide a novel insight into the mechanistic origin of Hg-mediated inflammation. It should be stressed that the effects reported herein were observed at exposure levels that do not cause appreciable cytotoxicity, demonstrating that this immunotoxic effect is a critical driver of toxicity. If eventually, these effects develop into a general activation of microglia, they could prompt a broader toxicological response serving as a primer for Hg’s deleterious neurotoxic events.

Most notably, up-regulating the activity of redox systems either through selenite or NAC supplementation mitigates the toxic effect of Hg^2+^ quite effectively, and the beneficial effects of other redox-active supplements are not to be disregarded also. This further reinforces the importance of an adequate nutritional status to minimize the toxicity resulting from Hg exposure in human populations at risk.

## Figures and Tables

**Table 1:** Primer sequences for mouse IL-1ß, iNOS, TNF-α and GAPDH.

mRNA Target	Forward Primer (5-3′)	Reverse Primer (5-3′)
Interleukin 1 (IL-1β)	TGCCACCTTTTGACAGTGATG	ATGTGCTGCTGCGAGATTTG
Nitric oxide synthase 2 (iNOS)	GTTCTCAGCCCAACAATACAAGA	GTGGACGGGTCGATGTCAC
Tumor Necrosis Factor (TNF-α)	TAGCCCACGTCGTAGCAAAC	GCAGCCTTGTCCCTTGAAGA
Glyceraldehyde-3-phosphate dehydrogenase (GAPDH)	GGAGAGTGTTTCCTCGTCCC	ATGAAGGGGTCGTTGATGGC

## Data Availability

The data presented in this study are available on request from the corresponding author.
